# Oncologic outcomes of segmentectomy for stage IA radiological solid-predominant lung cancer >2 cm in maximum tumour size

**DOI:** 10.1093/icvts/ivac246

**Published:** 2022-09-26

**Authors:** Aritoshi Hattori, Takeshi Matsunaga, Mariko Fukui, Kazuya Takamochi, Shiaki Oh, Kenji Suzuki

**Affiliations:** Department of General Thoracic Surgery, Juntendo University School of Medicine, Tokyo, Japan; Department of General Thoracic Surgery, Juntendo University School of Medicine, Tokyo, Japan; Department of General Thoracic Surgery, Juntendo University School of Medicine, Tokyo, Japan; Department of General Thoracic Surgery, Juntendo University School of Medicine, Tokyo, Japan; Department of General Thoracic Surgery, Juntendo University School of Medicine, Tokyo, Japan; Department of General Thoracic Surgery, Juntendo University School of Medicine, Tokyo, Japan

**Keywords:** Lung cancer, Stage IA, Segmentectomy, Ground-glass opacity

## Abstract

**OBJECTIVES:**

We aimed to compare the outcomes of segmentectomy with those of lobectomy in clinical-stage IA radiological solid-predominant non-small-cell lung cancer (NSCLC) >2 cm in maximum tumour size.

**METHODS:**

A retrospective review was performed for radiological solid-predominant NSCLC >2–3 cm in maximum tumour size with a ground-glass opacity component on thin-section computed tomography. Multivariable or propensity score-matched analyses were performed to control for confounders for survival. Overall survival (OS) was analysed using a Kaplan–Meier estimation.

**RESULTS:**

Of the 215 eligible cases, segmentectomy and lobectomy were performed in 46 and 169 patients. Multivariable analysis revealed that standardized uptake value (hazard ratio: 1.148, 95% confidence interval: 1.032–1.276, *P* = 0.011) was an independently significant prognosticators of OS, while the operative mode was not associated (hazard ratio: 0.635, 95% confidence interval: 0.132–3.049, *P* = 0.570). The 5 y-OS was excellent and did not differ significantly between segmentectomy and lobectomy (95.5% vs 90.2%; *P* = 0.697), which was also shown in the propensity score analysis (96.8% vs 94.0%; *P* = 0.406), with a median follow-up time of 5.2 years. Locoregional recurrence was found in 2 (4.3%) segmentectomy and 13 (7.7%) lobectomy (*P* = 0.443). In the subgroup analysis stratified by solid component size, the 5 y-OS was similar between segmentectomy and lobectomy in the c-T1b and c-T1c groups, respectively [c-T1b (*n* = 163): 94.1% vs 91.8%; *P* = 0.887 and c-T1c (*n* = 52): 100% vs 84.9%; *P* = 0.197].

**CONCLUSIONS:**

Segmentectomy showed similar oncological results compared to lobectomy in solid-predominant NSCLC with a ground-glass opacity component >2–3 cm in maximum tumour size. More prospective randomized trials are needed to adequately expand the indication of anatomic segmentectomy for early-stage NSCLC.

**Figure:**
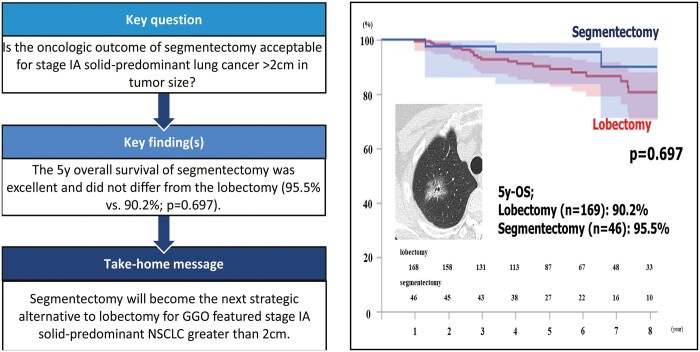

## INTRODUCTION

There is considerable controversy on the extent of parenchymal resection in the setting of stage I non-small-cell lung cancer (NSCLC). Since the study of the Lung Cancer Study Group in 1995, lobectomy has been considered a gold standard treatment for peripherally located small-sized NSCLC in patients who can tolerate anatomic resection [1]. However, over the last decade, there has been increasing evidence on the feasibility of the use of anatomic segmentectomy in early-stage NSCLC [2–5]. Furthermore, most recently, the pivotal result has been disclosed regarding a phase III randomized trial (JCOG0802/WJOG4607L) to confirm the non-inferiority of segmentectomy to lobectomy for survival, which demonstrated the benefit of segmentectomy with significant differences in overall survival (OS) [6]. These results indicate that segmentectomy might be a next standard surgical mode alternative to lobectomy for patients with small-sized (maximum tumour size ≤2 cm, consolidation-to-tumour ratio >0.5) peripheral c-stage IA NSCLC.

On the contrary, there are only a few data on the potential utility of anatomic segmentectomy for maximum tumour size >2 cm, especially in the radiological solid-predominant lesion [7–9]. In the 8th edition American Joint Committee on Cancer Lung Cancer Staging System, c-stage IA tumours are now divided into T1a-c lesions based on the size of the radiological solid component [10]. Under this new staging system, however, it has been reported that the presence of a ground-glass opacity (GGO) component is an important prognosticator regardless of the size of the solid component, which is considered as an oncologically distinct favourable entity [11–16]. Hence, it is theoretically expected to expand the surgical indication of segmentectomy while minimizing local recurrence and maximizing pulmonary functional reserve and survival outcomes for lung cancer with a GGO component.

In this regard, focusing on the presence of the GGO component as a promising radiological biomarker, we sought to compare the clinical outcomes of segmentectomy with those of lobectomy in clinical-stage IA radiological solid-predominant [0.5 < consolidation-to-tumour ratio (CTR) < 1.0] NSCLC >2 cm and up to 3 cm (i.e. >2–3 cm) in maximum tumour size, using data with an adequate follow-up period after surgery.

## MATERIALS AND METHODS

### Ethics statement

The medical records of each patient were retrospectively reviewed under a waiver of individual informed consent approved by the institutional review board of the Juntendo University School of Medicine, Tokyo, Japan (19-013).

### Study population

Between 2008 and 2018, we retrospectively reviewed the data of surgically resected NSCLC >2–3 cm in maximum tumour size, showing a radiological solid-predominant appearance on a thin-section CT scan. At our institute, the 8th edition of the TNM classification system were used for clinical staging [10]. There were no missing data for the variables examined in this study. The inclusion criteria were preoperative staging determined by thin-section CT and complete resection without preoperative chemotherapy and/or radiotherapy. Regarding the clinical nodal assessment, clinical-N0 indicated both nonenlarged lymph nodes (short axis <10 mm) on thin-section CT and no 18-fluorodeoxyglucose avidity on positron emission tomography (PET). Invasive modalities for mediastinal lymph node staging, such as mediastinoscopy or endobronchial ultrasound-guided transbronchial needle aspiration, were performed preoperatively to confirm the node-negative status in the case that the lymph nodes swelled on thin-section CT scan or positive on PET scan.

### Radiological evaluation of thin-section CT scan findings

For all patients, the findings of the preoperative thin-section CT scan were reviewed in detail by the authors (Aritoshi Hattori, Takeshi Matsunaga and Kenji Suzuki) and a radiology oncologist. Tumour size was determined preoperatively based on the thin-section CT findings. Furthermore, all tumours were subsequently evaluated to estimate the extent of GGOs by thin-section CT scan with a maximum slice thickness of 2-mm collimation. With regards to the institutional equipment from 2008 to 2018, 4-detector-row CT scanner or 16-detector-row CT scanner (Aquilion or Aquilion 16; Canon Medical Systems, Tochigi, Japan) was mainly used for the lung cancer diagnosis. The lung was photographed with a window level of −500 to −700 H and a window depth of 1000–2000 H as a ‘lung window’ and a window level of 30–60 H and a window depth of 350–600 H as a ‘mediastinal window’. GGO was defined as an area of slight and homogenous increase in density that did not obscure the underlying vascular markings. Furthermore, CTR was defined as the ratio of the maximum size of consolidation to the maximum tumour size on a thin-section CT scan [17]. In this study, lung cancer with a radiological solid-predominant appearance was defined as 1 with focal nodular opacity that contained both solid and GGO components (i.e. 0.5 < CTR < 1.0), excluding a pure-solid appearance (CTR = 1.0).

### Pathological evaluations

Lung adenocarcinomas were histologically classified according to the IASLC/ATS/ETS classification as adenocarcinoma *in situ*, minimally invasive adenocarcinoma and invasive adenocarcinoma [18]. Histological subtypes were classified according to the predominant subtype after a comprehensive histological subtyping, implying a semi-quantitative estimation of the percentage of different subtypes in increments of 5%. In this study, lepidic predominant adenocarcinoma was defined as a tumour that showed the lepidic component most frequently among invasive adenocarcinomas.

### Operation policy

Any surgical procedures were performed by small incision thoracotomy assisted by thoracoscope in our institute, maintaining a similar surgical quality for each case. A major lung dissection with systemic or selective lymph node dissection is essentially warranted for enrolled radiological solid-predominant NSCLC >2 cm. In contrast, segmentectomy is clinically indicated for patients in certain conditions, considering the tumour location allowed a sufficient surgical margin, CTR, or the existence of multifocal GGO lesions for which surgical treatment may be possible in the future. The sufficient margin distance between a tumour and the intersegmental plane was preoperatively evaluated by the three-direction thin-section CT scan (i.e. axial, sagittal and coronal view). If a sufficient surgical margin could not be surgically ensured, the resection line was extended to the adjacent segment of the lung to ensure an adequate resection margin. With regards to the postoperative adjuvant chemotherapy, Tegafur-Uracil (UFT) oral administration is recommended for p-stage I disease with invasive size >2 cm, and platinum-doublet chemotherapy was considered to stage II or more NSCLC.

### Follow-up policy

The routine follow-up evaluation included a physical examination, chest radiography, chest CT scan and blood tests including measurements of tumour markers every 6–12 months. If any symptom or sign of recurrence was observed, further evaluation was performed, including CT, brain magnetic resonance imaging and PET/CT to assess the locoregional or distant cancer recurrence. Locoregional recurrence was defined as occurrence within the residual same lobe and hilum or mediastinal lymph nodes, which was essentially diagnosed by cytological or histological confirmation based on biopsy or surgical resection.

### Statistics

In this study, the primary outcome of interest is to compare the long-term survival outcomes of segmentectomy with those of lobectomy in clinical-stage IA radiological solid-predominant (0.5 < CTR < 1.0) NSCLC >2 cm and up to 3 cm in maximum tumour size. Descriptive statistics for categorical variables were reported as frequencies and percentages, while continuous variables were reported as means (standard deviation) or medians (interquartile range), as appropriate. For categorical variables, comparisons between groups were made using Chi-square test or Fisher’s exact test. Continuous variables were compared using the Student’s *t*-test. Using SPSS Statistics 27 (IBM Inc., USA), Cox’s proportional hazard model was fitted to identify the clinicopathological factors affecting survival. Univariable and multivariable analyses, including all covariates, were performed using Cox’s proportional hazard model to adjust differences between groups by possible confounders. Preoperative comorbid status was evaluated using the Charlson comorbidity index and a score of ≥3 was defined as high comorbid status. Postoperative morbidity was evaluated according to the Common Terminology Criteria for Adverse Events ver. 5.0. In this study, ‘grade III or more’ was defined as a severe postoperative complication. Survival outcomes were estimated using the Kaplan–Meier method and compared using the log-rank test across the different groups. The date of surgical resection was set as the starting point; the date of death or survival follow-up was the end point for the calculation of OS. The difference was considered statistically significant when the *P*-value was <0.05 in the multivariable models. Furthermore, propensity score matching was used to control for confounders and reduce the prognostic imbalance conferred by selection bias. Clinicopathological variables listed in the tables were multiplied by a coefficient calculated from a logistic regression analysis. Variables included age, sex, operation side, smoking status, Charlson comorbidity index, tumour marker, respiratory function, maximum tumour size, solid component size, CTR, pathological-stage, histology, lymphatic or vascular invasion and postoperative chemotherapy. The sum of these values was taken as the propensity score for each patient. Patients who underwent segmentectomy and lobectomy with equivalent propensity scores were selected using a 1-to-1 greedy matching algorithm using the nearest neighbour without replacement, with a calliper width equal to 0.25 of the standard deviation of the logit of the propensity score [19]. Paired tests are used for comparisons within the matched sample, and standardized mean differences are used to assess the imbalance of covariates in the propensity score. Stratified log-rank test was used to compare actual survivals between matched groups. With regards to multiple comparisons in the subgroup analyses, *P*-value was adjusted by Bonferroni procedure. The method to report statistical data was instructed by the EJCTS and ICVTS guidelines [20].

## RESULTS

Of the 215 eligible patients, segmentectomy and lobectomy were performed in 46 and 169 patients, respectively, with a median follow-up period of 5.2 years. Table 1 demonstrates the demographic and clinicopathological variables between both groups. The segmentectomy group was older (*P* = 0.013), and the rate of a presence of additional ground-glass nodules was higher (*P* < 0.001) compared with the lobectomy group, while sex, Charlson comorbidity index or pulmonary function were not statistically different. Radiologically, the size of the solid component or CTR were not statistical different between the 2 study groups (*P* = 0.056, *P* = 0.077, respectively), showing similar ranges of solid component size or CTR between the 2 study groups. The number of dissected lymph nodes was significantly higher in the lobectomy group than the segmentectomy group (11 vs 6, *P* < 0.001), but the percentage of pathological nodal metastasis was not statistically different (6% vs 2%, *P* = 0.509).

**Table 1:** Clinicopathological characteristics based on the operative procedures

	Lobectomy	Segmentectomy	*P*-Value*
	(*n* = 169)	(*n* = 46)	
Age (years)	67.8 (9.6)	71.7 (9.5)	0.013
Sex (male)	77 (46)	23 (50)	0.59
Side (right)	112 (66)	24 (52)	0.08
Pack-year smoking	16.8 (22.4)	22.4 (31.3)	0.17
Charlson comorbidity index (high)	21 (12)	7 (15)	0.62
Additional ground-glass nodules (yes)	16 (10)	17 (37)	<0.001
Previous other cancer history (yes)	24 (14)	9 (20)	0.37
Carcinoembryonic antigen (ng/ml)	4.0 (4.5)	2.9 (2.1)	0.12
Maximum standardized uptake value	3.5 (3.1)	2.8 (1.7)	0.21
Forced expiratory volume in 1 s (%)	95.5 (17.9)	94.1 (17.6)	0.64
Vital capacity (%)	102.2 (16.3)	100.1 (17.7)	0.44
Diffusing capacity of carbon monoxide (%)	64.2 (18.6)	60.9 (17.7)	0.31
Maximum tumour size (mm)	25.1 (2.9)	24.5 (2.8)	0.22
Solid component size (mm)	18.3 (3.6)	17.1 (3.7)	0.06
Consolidation tumour ratio	0.73 (0.12)	0.69 (0.11)	0.08
Clinical stage (IA2/IA3)	128 (76)/41 (24)	35 (76)/11 (24)	0.96
Dissected lymph node number	11 (6-17)	6 (2-9)	<0.001
Extent of nodal dissection (hilar)	32 (19)	27 (59)	<0.001
p-N1/N2	6 (4)/4 (2)	1 (2)/0 (0)	0.51
Pathological stage (IA1/IA2/IA3/IB/IIA/IIB/IIIA)	27 (16)/83 (49)/36 (21)/12 (7)/3 (2)/5 (3)/3 (2)	17 (37)/14 (30)/7 (15)/7 (15)/1 (2)/0 (0)/0 (0)	<0.001
Pathological stage (stage IA)	146 (86%)	38 (83%)	0.52
Histology (Ad/Sq/others)	165 (98)/4 (2)/0 (0)	44 (96)/1 (2)/1 (2)	0.41
Dominant histology of adenocarcinoma (lepidic/acinar/papirally/solid/mucinous)	58 (34)/57 (34)/39 (23)/8 (5)/3 (2)	22 (48)/14 (30)/7 (15)/0 (0)/1 (2)	0.18
Lymphatic invasion (yes)	28 (17)	1 (2)	0.011
Vascular invasion (yes)	27 (16)	2 (4)	0.041
EGFR mutation (yes)	97 (57)	24 (52)	0.80
Operation time (min)	134 (112-166)	124 (106-145)	0.35
Bleeding amount (ml)	15 (10-25)	10 (6-20)	0.049
Morbidity (G3 or more)	18 (11)	5 (11)	0.97
Hospital stay (days)	8.2 (4.5)	8.0 (3.3)	0.76
Postoperative chemotherapy (yes)	61 (36)	4 (9)	<0.001
Postoperative recurrence			
Local recurrence	13 (7.7)	2 (4.3)	0.44
Distant (± local) recurrence	14 (8.3)	1 (2.2)	0.20
Cause of death			
Lung cancer	11 (6.5)	1 (2.2)	0.37
Other than lung cancer	9 (5.3)	4 (8.7)	0.40

Categorical data are shown as numbers (%) and continuous data are shown as mean (SD) if normally distributed and median (IQR) if not normally distributed.

* *P*-value in Chi-square test or Student's *t*-test.

IQR: interquartile range; SD: standard deviation

Table 2 shows the operative details of the segmentectomy. To secure the sufficient surgical margin for radiological solid-predominant NSCLC >2–3 cm in maximum tumour size, segmentectomy with resected three-segment was the most frequent, performed in 19 (41%) [left upper tri-segmentectomy, 16 (35%); basal segmentectomy, 3 (6%)], followed by S6 segmentectomy (*n* = 10, 22%). The other segmentectomies resected with <2 segments were indicated in 17 (37%) patients.

**Table 2:** Operative details of segmentectomy

	Segmentectomy (*n* = 46), *n* (%)	Lobectomy (*n* = 169), *n* (%)
Right upper lobe	8 (18)	69 (41)
S1 segmentectomy	3 (7)	
S2 segmentectomy	4 (9)	
S2 + S3a segmentectomy	1 (2)	
Right middle lobe	0 (0)	20 (12)
Right lower lobe	16 (35)	24 (14)
S6 segmentectomy	10 (22)	
S8 segmentectomy	4 (9)	
Basal segmentectomy	2 (4)	
Left upper lobe	19 (41)	39 (23)
S1 + 2 segmentectomy	1 (2)	
Upper tri-segmentectomy	16 (35)	
Lingular segmentectomy	2 (4)	
Left lower lobe	3 (6)	17 (10)
S6 segmentectomy	2 (4)	
Basal segmentectomy	1 (2)	

**Figure 1:**
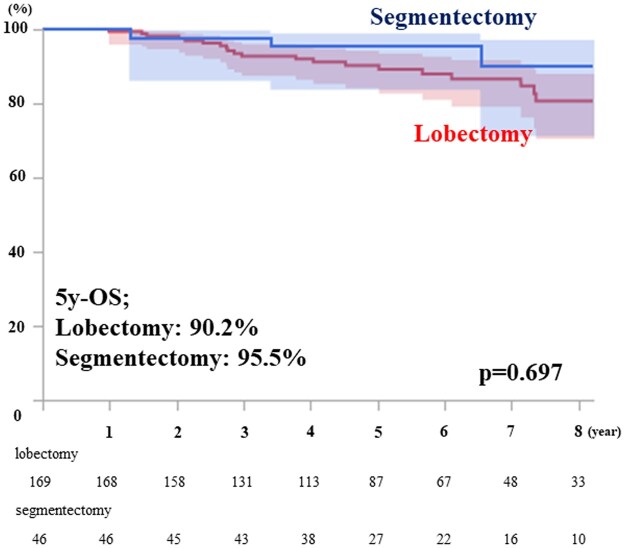
The survival curves are shown based on operative procedures in radiologically ground-glass opacity featured solid-predominant non-small-cell lung cancer >2–3 cm in maximum tumour size.

**Figure 2:**
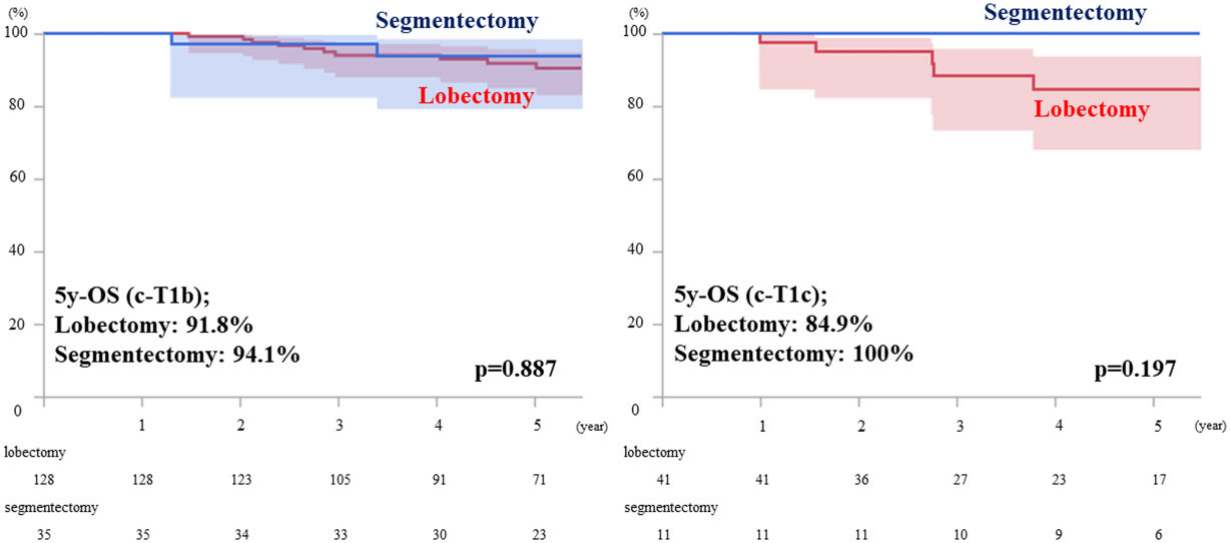
The results of the overall survival are stratified by the radiological solid component size. Left Figure is the result of c-T1b lesion (n=163), and right Figure is the result of c-T1c lesion (n=52).

The result of the Cox proportional hazard model for OS is shown in Table 3. A multivariable analysis revealed that maximum standardized uptake value (hazard ratio: 1.148, 95% confidence interval: 1.032–1.276, *P* = 0.011) was an independently significant clinical prognosticator of OS, while the operative mode was not associated with the survival outcome (hazard ratio: 0.635, 95% confidence interval: 0.132–3.049, *P* = 0.574). Accordingly, the survival outcomes were excellent despite the operative modes, which were not significantly different between segmentectomy and lobectomy (Fig. 1, 5 y-OS: 95.5% vs 90.2%, *P* = 0.697). In Table 1, the percentage of lung cancer recurrence was shown, which was similar between lobectomy versus segmentectomy [loco-regional, 13 (8%) vs 2 (4%), *P* = 0.443; distant, 14 (8%) vs 1 (2%), *P* = 0.201]. The details of loco-regional recurrence after segmentectomy were as follows: intrapulmonary metastasis and mediastinal nodal metastasis after lingular segmentectomy (solid component size 21 mm, CTR = 81%) and pleural dissemination after S6 segmentectomy (solid component size 18 mm, CTR = 78%). Regarding the cause of death, both lung cancer death and the other cause of death were not significantly different. There was no 90-day mortality in this cohort. Furthermore, clinicopathological characteristics in the subgroup analysis stratified by the AJCC 8th clinical T staging (i.e. c-T1b and c-T1c lesions) were demonstrated in Supplementary Material, Table S1. According to the subgroup analysis, OS was also similar between segmentectomy and lobectomy in the c-T1b and c-T1c groups, respectively [Fig. 2a, c-T1b (*n* = 163); 94.1% vs 91.8%, *P* = 0.887, Fig. 2b, c-T1c (*n* = 52); 100% vs 84.9%, *P* = 0.197].

**Table 3:** Cox proportional hazard model for the overall survival

	Univariable		Multivariable	
Variable	HR (95% CI)	*P*-Value*	HR (95% CI)	*P*-Value*
Charlson comorbidity index (high)	0.583(0.250-1.359)	0.21		
Carcinoembryonic antigen (ng/ml)	1.131(1.089-1.185)	<0.001	1.075(0.982-1.178)	0.12
Maximum standardized uptake value	1.193(1.097-1.297)	<0.001	1.148(1.032-1.276)	0.011
Maximum tumour size (mm)	1.101(0.962-1.260)	0.16		
Solid component size (mm)	1.079(0.970-1.200)	0.16	1.022(0.871-1.197)	0.79
Operative mode (segmentectomy)	0.823(0.309-2.195)	0.70	0.635(0.132-3.049)	0.57
Extent of lymph nodal dissection (hilar)	1.791(0.784-4.092)	0.17		

* *P*-value in the Cox proportional hazard model.

CI: confidence interval; HR: hazard ratio.

**Figure 3:**
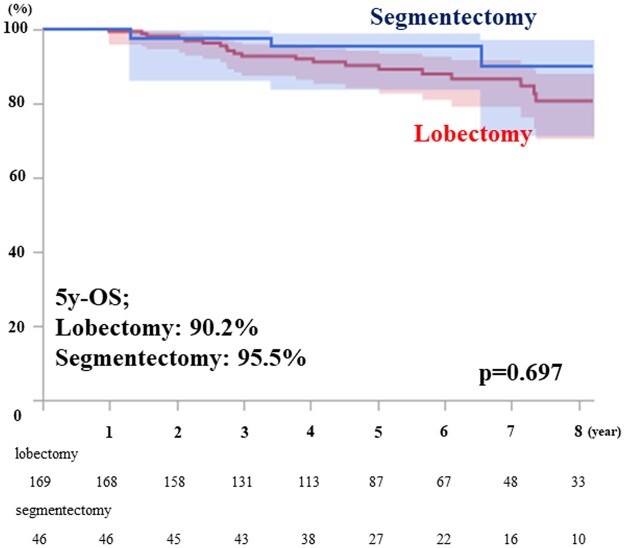
The survival curves are shown for selected patients after propensity score-matched analysis.

Supplementary Material, Table S2 shows the results of the propensity score-matched analysis in which 35 matched pairs between segmentectomy and lobectomy were assessed. After matching, each of the clinicopathological variables was well balanced between the 2 study arms. Even in the propensity score-matched cohort, no significant survival difference was observed between the segmentectomy and lobectomy (Fig. 3a, 5 y-OS: 94.0% vs 96.8%, *P* = 0.406).

## DISCUSSION

While the several evidences are accumulated in recent years regarding the feasible oncologic outcomes of segmentectomy for small-sized peripheral NSCLC 2 cm or less in size, lobectomy is still considered a main surgical strategy for NSCLC >2 cm in maximum tumour size from the point of locoregional cancer control. However, recent institutional reports have noticed a favourable prognosis of NSCLC with a component of GGO, regardless of the size of the solid component [11–16]. Therefore, focusing on the presence of the GGO component as a provocative radiological biomarker, our objective was to compare the outcomes of segmentectomy with those of lobectomy in radiological solid-predominant (0.5 < CTR < 1.0) NSCLC >2–3 cm in maximum tumour size, to see if it is possible to adequately expand the surgical indication of segmentectomy. As a result, segmentectomy not only demonstrated excellent oncologic outcomes, but loco-regional cancer control was fully acceptable compared to lobectomy for these entities. In the future, segmentectomy to preserve lung parenchyma will likely become the next novel strategic alternative to lobectomy for GGO featured stage IA solid-predominant NSCLC >2 cm.

There is no doubt that the size of the solid component better reflects the malignant potential than the overall tumour size in lung adenocarcinoma, as the size of the solid component excluding the GGO was assigned to the 8th edition of clinical T classification [10]. In contrast, the concept of the prognostic importance of a GGO component in early-stage NSCLC has been studied on a nationwide level [21]. The most important clinical implication is that part-solid lung adenocarcinoma demonstrates favourable oncological outcomes, being >90%, regardless of the size of the solid component in cases where the tumour showed a GGO component [15, 21, 22]. In contrast, radiologically solid tumours without any GGO component reveal unfavourable biology and a significantly dismal prognosis than lung cancer with a GGO component. Due to the distinct clinicopathological and oncological characteristics based on the presence of the GGO component, we excluded a radiologically pure-solid NSCLC in this analysis to eliminate the potential risk of loco-regional cancer recurrence.

In the circumstance, not only were the entire 5 y-OS and both the c-T1b and c-T1c subgroups similar between segmentectomy and lobectomy, but also their survival outcomes of segmentectomy were excellent, showing >90%, provided that the tumour showed radiologically solid-predominant appearance with a GGO component in patients with NCSCL >2–3 cm in maximum tumour size. Furthermore, lung cancers presenting with GGO components often have more than one nodule detected on their CT scans [23, 24]. It is also notable that patients cured of their initial lung cancer are more likely to develop second primary lung cancer than the general population, which has been estimated to have an annual risk of developing second primary lung cancer is as high as 3% [25]. In fact, the rate of additional In that context of multiple nodules and potential future cancers, parenchyma-sparing surgical procedures may also become increasingly important for long-term lung preservation. Although further investigation is necessary, these lesions might be the next candidates for segmentectomy, which contribute to more extensive treatment opportunity for cancer relapse or second primary lung cancer, or possibly for other cancers, resulting in longer OS.

Concerning the proper indication of segmentectomy for solid-predominant NSCLC larger than 2 cm, we should pay special attention to avoiding locoregional cancer recurrence. Securing a sufficient surgical margin would be a crucial matter of concern to prevent inadequate cancer control. However, the frequency of locoregional recurrence was low, and surgical stump recurrence was never experienced in the current study. These excellent oncologic results might be explained by the clinical evidence that several pathological factors, i.e. lymphatic or vascular invasion, visceral pleural invasion or spread throughout the alveolar space, are not related to an adverse prognosis in stage I NSCLC provided that the tumour showed a GGO component [26–28]. To ensure a sufficient surgical margin, it is expected to develop a novel three-dimensional technology to confirm enough surgical margins and demonstrate the appropriate intersegmental plain to expand the proper surgical indication for segmentectomy in the future. Furthermore, hilar lymph node dissection might not be enough in the segmentectomy compared to the lobectomy [6], which resulted in the different number of dissected lymph node or extent of nodal dissection between the 2 study arms in the current study. However, these differences were not influenced on the survival or locoregional recurrence. It is debateable regarding the proper extent of nodal dissection for solid-predominant NSCLC larger than 2 cm. Theoretically, however, radiological part-solid tumours are less likely to spread to regional lymph nodes, and the oncological characteristics are less likely to be invasive, which are a potentially better candidate for segmentectomy despite the tumour size.

### Limitations

There were some limitations to this study. First, this study was based on a single-institution Japanese database with a relatively small sample size of the segmentectomy, because the lobectomy has been mainly indicated as a standard surgical policy for the study population. Hence, the probability of type II error might not be eliminated, and it is difficult to control differences for possible confounders. Furthermore, the 2 groups are not fully comparable as the indication of 1 technique over the other is based on different clinical and anatomical considerations. Hence, the cautions are still necessary to conclude the efficacy and safety of anatomical segmentectomy for the study population because it is impossible to compare them of both procedures given that they were performed in patients with different characteristics. Second, the median follow-up period was longer than 5 years; however, further observation is necessary to assess the oncological impact of the segmentectomy for GGO featured NSCLC. Third, our study was subject to the inherent biases of a retrospective study, the most important of which is the selection bias in the allocation of treatment. The propensity score-matched analysis in the current study may have been a potential remedy to control bias between the treatment groups. However, proper comparisons are difficult to make in a single-centre experience due to the relatively small cases. Furthermore, there is increasing interest in this area in an era in which treatment for early-stage NSCLC is being developed. Therefore, the clinical implications of the analysis to our daily practice will be immense. It is necessary to confirm the efficacy of segmentectomy for larger sized lung cancer with a GGO component based on a randomized prospective study.

## CONCLUSION

In conclusion, segmentectomy demonstrated feasible oncological results as well as those of lobectomy for radiological solid-predominant NSCLC with a GGO of >2–3 cm in maximum tumour size. Further prospective randomized trials are warranted to corroborate the expansion of the surgical indication for anatomic segmentectomy.

## SUPPLEMENTARY MATERIAL

Supplementary material is available at *ICVTS* online.

## Supplementary Material

ivac246_Supplementary_Data

## Data Availability

The data underlying this article cannot be shared publicly due to the privacy of individuals that participated in the study.

## ABBREVIATIONS

CTR: Consolidation-to-tumour ratio
GGO: Ground-glass opacity
NSCLC: Non-small-cell lung cancer
OS: Overall survival
PET: Positron emission tomography
