# Supplementary material for: Oncologic outcomes of segmentectomy for stage IA radiological solid-predominant lung cancer >2 cm in maximum tumour size
Source: Interact Cardiovasc Thorac Surg. 2022 Sep 26;35(6):ivac246. doi: 10.1093/icvts/ivac246 (PMC9725180; doi:10.1093/icvts/ivac246)
Supplement: ivac246_Supplementary_Data [file ivac246_supplementary_data.doc]

**Supplemental Table 1. Clinicopathological characteristics in the subgroups stratified by the clinical T staging**

|  | Clinical T1b (n=163) | |  |  | Clinical T1c (n=52) | |  |
| --- | --- | --- | --- | --- | --- | --- | --- |
| Clinical factors | Lobectomy  (n=128 (79%)) | Segmentectomy  (n=35 (21%)) | P* |  | Lobectomy  (n=41 (79%)) | Segmentectomy  (n=11 (21%)) | P* |
| Age (y) | 67.8(9.9) | 72.7(8.7) | 0.026 |  | 67.7(8.9) | 68.6(8.4) | 0.77 |
| Sex (male) | 55(43) | 17(51) | 0.37 |  | 22(54) | 5(46) | 0.63 |
| Side (right) | 86(67) | 17(49) | 0.09 |  | 26(63) | 7(74) | 0.99 |
| Pack-year smoking | 16.3(22.0) | 22.8(32.4) | 0.17 |  | 18.3(24.2) | 21.1(28.8) | 0.75 |
| Charlson comorbidity index (high) | 12(9) | 5(14) | 0.40 |  | 9(22) | 2(18) | 0.79 |
| Multiple lung cancers (yes) | 12(9) | 10(29) | 0.006 |  | 4(10) | 7(64) | <0.001 |
| Previous other cancer history (yes) | 18(14) | 8(23) | 0.21 |  | 6(15) | 1(9) | 0.63 |
| Carcinoembryonic antigen (ng/ml) | 3.9(4.5) | 3.1(2.2) | 0.12 |  | 4.4(4.9) | 2.4(1.6) | 0.18 |
| Maximum standardized uptake value | 3.0(2.7) | 2.9(1.9) | 0.21 |  | 5.1(3.9) | 2.6(1.1) | 0.06 |
| Forced expiratory volume in 1 sec (%) | 96.9(18.6) | 93.7(19.9) | 0.92 |  | 90.1(14.5) | 95.4(7.4) | 0.32 |
| Vital capacity (%) | 103.2(17.1) | 100.2(18.0) | 0.44 |  | 99.0(12.8) | 99.6(14.4) | 0.90 |
| Diffusing capacity of carbon monoxide (%) | 65.8(18.4) | 60.4(18.9) | 0.31 |  | 59.5(18.7) | 62.6(12.9) | 0.66 |
| Maximum tumor size (mm) | 24.3(2.8) | 23.6(2.2) | 0.24 |  | 27.4(1.9) | 27.5(2.3) | 0.95 |
| Solid component size (mm) | 16.7(2.4) | 15.5(2.3) | 0.11 |  | 23.0(2.1) | 22.3(2.1) | 0.28 |
| Consolidation tumor ratio | 0.69(0.11) | 0.66(0.08) | 0.15 |  | 0.84(0.08) | 0.81(0.09) | 0.31 |
| Dissected lymph node number | 12±8 | 7±6 | <0.001 |  | 11±7 | 8±5 | 0.13 |
| Extent of nodal dissection (hilar) | 23(18) | 20(57) | <0.001 |  | 9(22) | 7(64) | 0.016 |
| p-N1/N2 | 5(4)/3(2) | 1(3)/0(0) | 0.44 |  | 1(2)/1(2) | 0(0) | 0.46 |
| pathological-stage (stage IA) | 115(90) | 29(83) | 0.25 |  | 31(76) | 9(82) | 0.66 |
| Histology (Adenocarcinoma) | 126(98) | 33(94) | 0.16 |  | 39(95) | 11(100) | 0.46 |
| Lepidic predominant adenocarcinoma (yes) | 50(39) | 16(46) | 0.48 |  | 8(20) | 6(55) | 0.06 |
| Lymphatic invasion (yes) | 20(16) | 1(3) | 0.09 |  | 8(20) | 0(0) | 0.11 |
| Vascular invasion (yes) | 15(12) | 2(6) | 0.30 |  | 12(29) | 0(0) | 0.08 |
| EGFR mutation (yes) | 75(59) | 17(49) | 0.57 |  | 22(54) | 7(64) | 0.83 |
| Operation time (min) | 139.5(40.7) | 132.0(42.7) | 0.345 |  | 141.6(40.7) | 138.1(54.6) | 0.82 |
| Bleeding amount (ml) | 24.1(24.3) | 15.1(13.1) | 0.10 |  | 33.6(63.4) | 15.3(12.7) | 0.35 |
| Morbidity (G3 or more) | 13(10) | 4(11) | 0.83 |  | 5(12) | 1(9) | 0.78 |
| Hospital stay (day) | 7.9(3.5) | 8.2(3.6) | 0.61 |  | 9.3(6.6) | 7.4(2.3) | 0.33 |
| Postoperative chemotherapy (yes) | 43(34) | 3(9) | 0.008 |  | 18(44) | 1(9) | 0.07 |

Categorical data are shown as numbers (%) and continuous data as mean (SD) if normally distributed, and median (IQR) if not normally distributed.

* P-value in Chi square test or Student's t-test

**Supplemental Table 2. Propensity-score matched comparison**

|  | **Lobectomy**  **(n=35)** | **Segmentectomy**  **(n=35)** | **P*** | **SMD**** |
| --- | --- | --- | --- | --- |
| Age (y) | 72.1(7.4) | 72.8(8.5) | 0.869 | 0.08 |
| Sex (male) | 13(37) | 16(46) | 0.467 | 0.18 |
| Side (right) | 22(63) | 21(60) | 0.806 | 0.06 |
| Pack-year smoking | 15.4(21.7) | 18.8(22.5) | 0.516 | 0.14 |
| Charlson comorbidity index (high) | 5(14) | 6(17) | 0.743 | 0.08 |
| Multiple lung cancers (yes) | 8(23) | 9(26) | 0.780 | 0.06 |
| Previous other cancer history (yes) | 9(26) | 8(23) | 0.780 | 0.06 |
| Carcinoembryonic antigen (ng/ml) | 2.7(1.2) | 3.2(2.2) | 0.296 | 0.16 |
| Maximum standardized uptake value | 3.4(3.8) | 3.0(1.8) | 0.633 | 0.12 |
| Forced expiratory volume in 1 sec (%) | 91.5(19.5) | 92.9(17.1) | 0.744 | 0.07 |
| Vital capacity (%) | 98.5(15.6) | 99.2(16.1) | 0.831 | 0.08 |
| Diffusing capacity of carbon monoxide (%) | 60.3(13.5) | 59.5(19.2) | 0.869 | 0.05 |
| Maximum tumor size (mm) | 23.8(2.6) | 24.5(2.8) | 0.308 | 0.25 |
| Solid component size (mm) | 17.2(3.4) | 17.0(4.0) | 0.822 | 0.05 |
| Consolidation tumor ratio | 0.72(0.12) | 0.69(0.11) | 0.244 | 0.24 |
| Dissected lymph node number | 8(6) | 8(6) | 0.778 | 0 |
| Extent of nodal dissection (hilar) | 16(46) | 18(51) | 0.632 | 0.10 |
| p-N1/N2 | 0(0) | 0(0) | 1.000 | 0 |
| pathological-stage (stage IA) | 31(89) | 31(89) | 1.000 | 0 |
| Histology (adenocarcinoma)) | 34(97) | 33(94) | 0.555 | 0.06 |
| Lepidic predominant adenocarcinoma (yes) | 15(43) | 16(46) | 0.810 | 0.06 |
| Lymphatic invasion (yes) | 1(3) | 1(3) | 1.000 | 0 |
| Vascular invasion (yes) | 3(9) | 2(6) | 0.643 | 0.10 |
| Operation time (min) | 136.1(46.7) | 134.6(46.1) | 0.896 | 0.03 |
| Bleeding amount (ml) | 19(23) | 16(14) | 0.560 | 0.15 |
| Morbidity (G3 or more) | 4(11) | 4(11) | 1.000 | 0 |
| Postoperative chemotherapy (yes) | 4(11) | 3(9) | 0.690 | 0.07 |

Categorical data are shown as numbers (%) and continuous data as mean (SD) if normally distributed, and median (IQR) if not normally distributed

* P-value in chi-squared test or Student's t-test

SMD**=standardized mean difference
